# 3-Year Post-Transcatheter Aortic Valve Replacement (TAVR) Restenosis: A Rare Complication

**DOI:** 10.7759/cureus.53515

**Published:** 2024-02-03

**Authors:** Ijeoma Orabueze, Olushola Ogunleye, Mark Harrison

**Affiliations:** 1 Internal Medicine, Vassar Brothers Medical Center, Poughkeepsie, USA

**Keywords:** early valve dysfunction, edwards sapiens valve, pv thrombosis, savr, tavr

## Abstract

The pathophysiology of aortic valve stenosis is degenerative and calcific. It may be exacerbated by atherosclerotic processes characterized by lipid accumulation and inflammation. When the stenosis gets hemodynamically severe, the valves would need to be replaced. They could be replaced using mechanical or bioprosthetic heart valves. Balloon-expandable (BE) transcatheter heart valves (THVs) were compared to the self-expanding (SE) THVs and it was found that the rate of bioprosthetic valve failure was low over a five-year period. We present the case of a 70-year-old female who presented with worsening shortness of breath three years after transcatheter aortic valve replacement and was found to have early valve degeneration.

## Introduction

The two types of artificial heart valves are mechanical (MHV) and bioprosthetic (BHV). BHVs can be implanted surgically or via the transcatheter approach [1]. Since its approval in the United States in 2011, transcatheter aortic valve replacements (TAVRs) have provided an alternative therapy when the mortality rate of surgical aortic valve replacement (SAVR) was high [2]. The Placement of Aortic Transcatheter Valves (PARTNER-2A) trial found that the incidence of aortic valve reintervention was 0.7% and 3.2% at 2 years and 5 years respectively [3]. Of the 3.2%, 10% were due to aortic valve stenosis indicating the rarity of early bioprosthetic valve deterioration [3].

## Case presentation

We present a 70-year-old female with a medical history of hypertension, hyperlipidemia, severe aortic stenosis requiring TAVR, drop attacks, significant tobacco use, chronic neck pain, and low back pain with prior cervical spine surgery on chronic opiates. She had a transfemoral transcatheter placement of a 23 mm Sapien 3 valve (Edwards Lifesciences Corp., Irvine, CA, USA) three years prior. On postoperative day 1, she was found to have a slight increase in aortic valve mean gradient on transthoracic echocardiogram (TTE) to 18.6 mmHg, aortic peak velocity of 2.8 m/s, aortic valve area of 1.8 cm² but no paravalvular leak and was started on rivaroxaban to prevent valve leaflet thrombosis and reduce the transvalvular gradient. Follow-up TTE a month later revealed an aortic valve mean gradient of 17.8 mmHg. Rivaroxaban was stopped a year later, and aspirin resumed as her aortic valve mean gradients remained stable; her aortic valve mean gradient was 19 mmHg, peak velocity was 2.9 m/s, and valve area 1.4 cm². 

**Figure 1:**
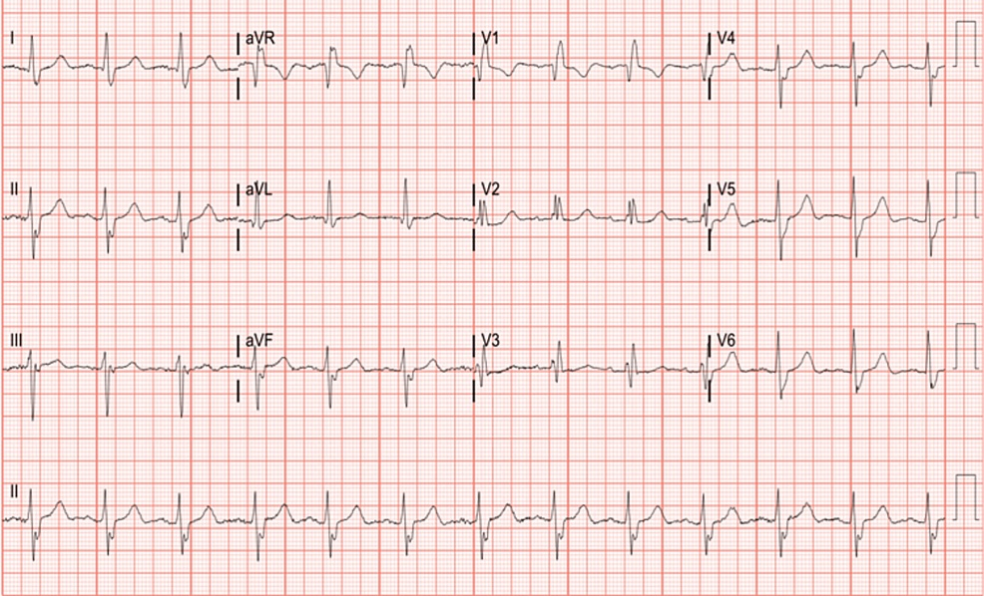
EKG showing normal sinus rhythm, right bundle branch block (RBBB), left anterior fascicular block (LAFB) (unchanged from prior EKGs).

**Figure 2:**
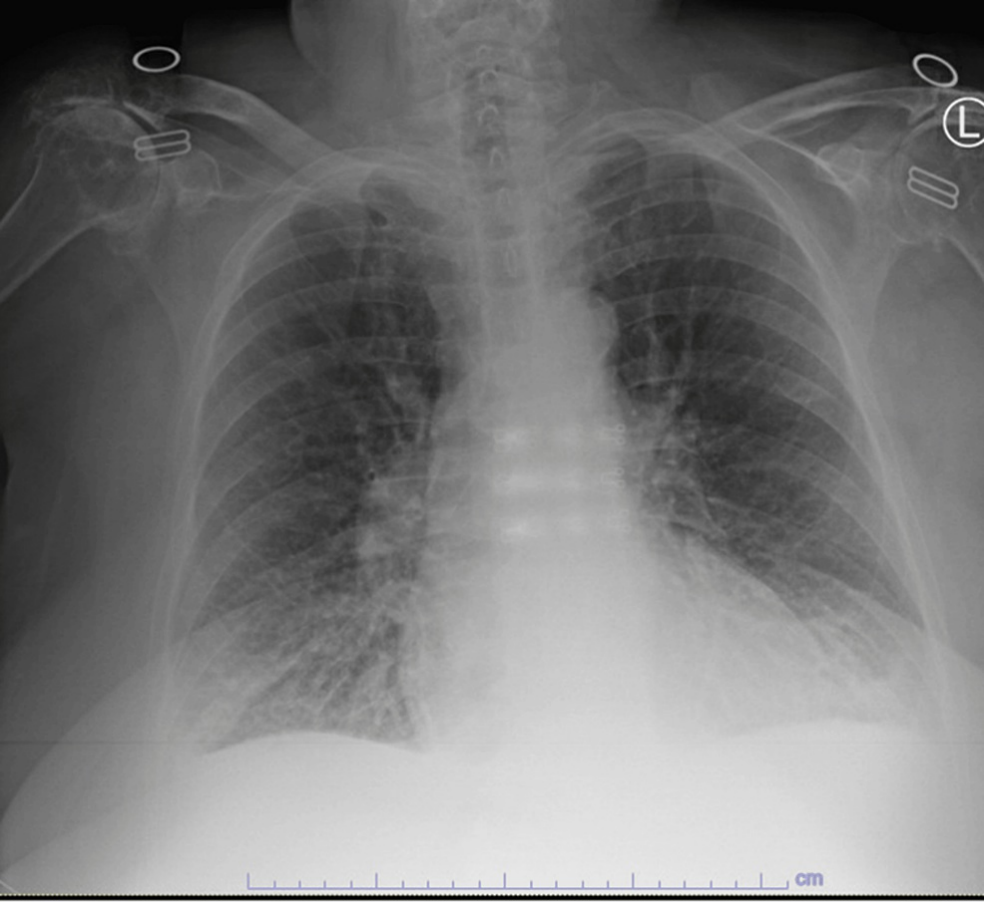
Chest x-ray showing mild patchy increased opacities in the lung bases possibly representing mild edema or atypical pneumonia in the appropriate clinical setting.

Three years post-op, the patient developed sudden onset shortness of breath, preventing her from walking up the stairs or down the hallway without stopping to rest. This prompted her to visit her cardiologist, who noted a loud new-onset murmur on examination, and she was sent to the emergency department. An electrocardiogram revealed a normal sinus rhythm, right bundle branch block, and left anterior fascicular block (Figure 1). A chest x-ray revealed bilateral bibasilar lung opacities. Her NT-ProBNP was 13,838 pg/ml (normal range <= 299 pg/ml) (Figure 2).

**Figure 3:**
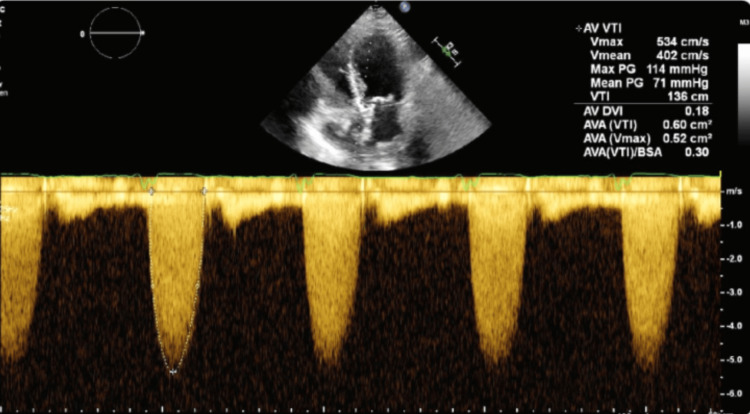
Transthoracic echocardiogram showing velocity across the bioprosthetic valve of 5.4 m/s, a mean gradient of 71 mmHg, and an aortic valve area of 0.5 cm.

**Figure 4:**
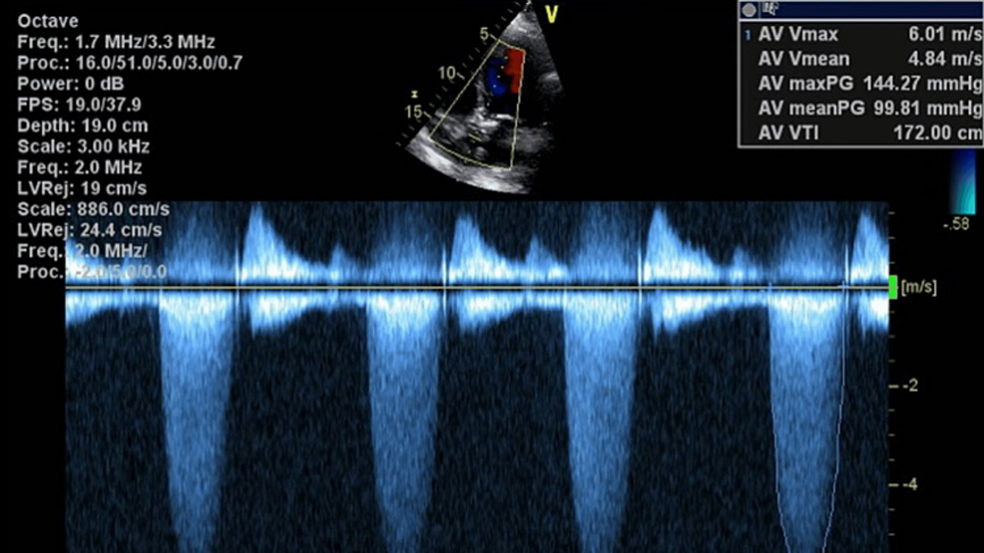
Transthoracic echocardiogram showing the aortic peak velocity of 6.1 m/s, the highest AV mean gradient of 99.8 mmHg, and the AV area calculated at 0.37 cm². The AV velocity ratio/Dimensionless Index is 0.14. There is severe prosthetic aortic stenosis. AV: Aortic valve.

TTE revealed a mean pressure gradient of 71 mmHg and an aortic peak velocity of 5.4 m/s (Figure 3). The TTE prior to this, was done a year earlier and showed aortic peak velocity of 2.9 m/s and valve mean gradient of 18.1 mmHg. The patient was started on a furosemide drip and heparin drip and transitioned to warfarin and clopidogrel due to high clinical suspicion of valve leaflet thrombosis. Subsequent TTE showed worsening findings despite treatment, with an aortic peak velocity of 6.1 m/s, severely increased aortic valve mean gradient of 99.8 mmHg, and aortic valve area of 0.37 cm² (Figure 4). She underwent a surgical aortic valve replacement and was placed on warfarin after the surgery. The culture of the explanted TAVR valve grew *Microbacterium paraoxydans*, which was later determined to be a contaminant, with endocarditis ruled out. The pathology report showed valvular tissue with fibrinoid necrosis, hyaline degeneration, and extensive calcifications.

## Discussion

TAVR placement has been associated with a 7.8% risk of all-cause 30-day mortality [2]. It has also been associated with a 3.2% risk of stroke, a 7.4% risk of moderate to severe residual aortic regurgitation, a 15.6% risk of life-threatening bleeding, and a risk of permanent pacemaker implant [2]. Valve embolization, coronary occlusion, acute kidney injury, and prosthetic dysfunction have also been associated with TAVR placement [2]. 

BHVs are less thrombogenic than MHVs and have a 1% risk of symptomatic thrombosis after placement [4]. The prosthetic valves can be implanted surgically or via the transcatheter approach. The surgical BHVs consist of bovine or porcine tissue and are mounted on a frame or stent covered with fabric [1]. The transcatheter BHVs consist of porcine or bovine pericardial tissue, are tri-leaflet, and are mounted on a self-expandable or balloon-expandable frame [1]. The Sapien 3 valve (Edwards Lifesciences Corp., Irvine, CA, USA) used in our patient is a bovine aortic tri-leaflet valve attached to a metallic scaffold. The stent is expandable and is manually crimped onto the balloon immediately before deployment. The CHOICE trial performed by Abdel-Wahab et al. compared the balloon-expandable (BE) valve to the self-expanding (SE) valve over five years and concluded that the rate of valve failure and device performance was not significantly different between both groups (4.1% vs. 3.4%; p = 0.63) [5]. This means the bioprosthetic valve should typically last at least five years [5].

Clinical valve dysfunction post-TAVR may not only be due to thrombus formation; rare occurrences like early valve degeneration can occur [1]. Like in our patient, it can present with clinically significant New York Hearth Association (NYHA) II-IV heart failure symptoms and hemodynamic instability. There are four main causes of valve dysfunction: valve thrombus, pannus formation, degeneration, and endocarditis with vegetation [1]. Therapeutic anticoagulation is useful for the management of subclinical leaflet thrombosis which is characterized by the presence of reduced leaflet motion and hypo-attenuated leaflet thickening. However, our patient failed to improve on heparin drip and warfarin, making valve thrombosis less likely.

The pathophysiology of prosthetic valve (PV) thrombosis involves platelet aggregation and deposition, thrombin generation, and clot formation while that of pannus formation involves thrombin generation, fibrin deposition, fibroblast proliferation, collagen deposition, and neo-angiogenesis [1]. Due to the absence of a thrombus on TTE and failure to improve on anticoagulation, pannus formation vs early valve degeneration was regarded as the more likely etiologic process. However, the only definitive diagnosis would have been after surgical intervention. Since no thrombus or pannus formation was noted during surgery, and in the absence of clinical evidence of endocarditis, it was determined that our patient most likely had severe valve deterioration of unclear etiology. This was confirmed by the pathology report a few weeks later.

Lack of anticoagulation therapy, use of a smaller valve, a valve-in-valve procedure, and high BMI are some factors that have been associated with an increased risk of valve dysfunction after TAVR [6]. The predisposing factors to her early valve degeneration, despite appropriate anticoagulation with rivaroxaban in the first year and aspirin thereafter, have yet to be ascertained. However, some of the risk factors for early valve degeneration in this patient could be mechanical stress due to mounting the valve tissue within the transcatheter design, leaflet crimping prior to valve delivery, remnant native valve calcification which may negatively impact the valve geometry, and under or over-expansion of the stent frame [7].

## Conclusions

We highlight this case to draw attention to early post-transcatheter aortic valve replacement (TAVR) bioprosthetic valve dysfunction necessitating surgical aortic valve replacement. Some risk factors could be mechanical stress due to mounting the valve tissue within the transcatheter design, leaflet crimping before valve delivery, remnant native valve calcification negatively impacting the valve geometry, and under- or over-expansion of the stent frame. We also draw attention to the need for clear antithrombotic therapy guidelines post-TAVR.
